# Monitoring *EGFR*-lung cancer evolution: a possible beginning of a “methylation era” in TKI resistance prediction

**DOI:** 10.3389/fonc.2023.1137384

**Published:** 2023-04-20

**Authors:** Federico Pio Fabrizio, Angelo Sparaneo, Lucia Anna Muscarella

**Affiliations:** Laboratory of Oncology, Fondazione IRCCS Casa Sollievo della Sofferenza, San Giovanni Rotondo (FG), Italy

**Keywords:** lung cancer, tyrosine kinase inhibitors, resistance, methylation, epigenetic markers

## Abstract

The advances in scientific knowledge on biological therapies of the last two decades have impressively oriented the clinical management of non-small-cell lung cancer (NSCLC) patients. The treatment with tyrosine kinase inhibitors (TKIs) in patients harboring Epidermal Growth Factor Receptor (*EGFR*)-activating mutations is dramatically associated with an improvement in disease control. Anyhow, the prognosis for this selected group of patients remains unfavorable, due to the innate and/or acquired resistance to biological therapies. The methylome analysis of many tumors revealed multiple patterns of methylation at single/multiple cytosine-phosphate-guanine (CpG) sites that are linked to the modulation of several cellular pathways involved in cancer onset and progression. In lung cancer patients, ever increasing evidences also suggest that the association between DNA methylation changes at promoter/intergenic regions and the consequent alteration of gene-expression signatures could be related to the acquisition of resistance to biological therapies. Despite this intriguing hypothesis, large confirmatory studies are demanded to consolidate and finalize many preliminary observations made in this field. In this review, we will summarize the available knowledge about the dynamic role of DNA methylation in *EGFR*-mutated NSCLC patients.

## 1 Introduction

The paradigm of cancer has evolved in the last years and conveyed into the concept of cancer “epigenome”, strictly linked to cancer “genome” (1). Many epigenetic regulatory players are involved in the transcription modulation of multiple tumor suppressor genes (TSG); DNA methylation, histone modifications, aberrant expression of microRNAs (miRNAs) and long non-coding RNAs (lncRNAs) participate in many neoplastic steps, such as dysregulation of cell growth, malignant cell transformation, invasion and metastatization (2–5).

Among all epigenetic alterations, DNA methylation represents one of the most studied chemical modifications in human disease. It occurs when methyl groups are covalently attached to the carbon at 5’ position of the cytosine residue of DNA by the DNA methyltransferase (DNMT) enzymes (6, 7).

The recent implementation of high-throughput approaches for methylation analysis gives a more detailed and dynamic overview of the DNA methylation perturbation in human disease and provides new important insights on the understanding of both temporal and spatial related gene expression modification and chromatin remodeling (8–10). This enhances, by consequence, to better explain the role of this class of epigenetic changes in cancer biology, unveiling novel epigenetic predictive and prognostic molecular biomarkers for neoplastic disease monitoring and outcome prediction in patients (11, 12).

The human cancer cells are characterized by the presence of a complex aberrant methylation signature, which takes place either as a hypo- or hyper-methylation events at single interspersed CpGs and/or CpG islands located both in the promoter and intergenic regions of genes. These epigenetic modifications may represent an early event in cancer development and progression, as well as they could cooperate with genetic lesions to guide the heterogeneity of response/resistance to therapies in patients (13, 14).

In lung cancer, the association between aberrant methylation profiles and resistance to anti-EGFR therapy is still understudied. More attention is required, since changes in methylation levels may help to explain the observed heterogeneity of lung tumor response to multiple targeted therapies (14). Here we detailed and updated the most recent advances in DNA methylation modifications linked to TKI resistance mechanisms in *EGFR*-mutated patients and their related cellular pathways (15, 16).

Scientific evidences on the role of miRNA signature alterations as players in TKI of EGFR resistance was also briefly discussed. All available scientific evidences about the prognostic value of epigenetic alterations as primary/intrinsic and secondary/acquired mechanisms of resistance were summarized. Publications in English language, peer-reviewed international journals were identified on PubMed. All scientific knowledges were updated until October 2022.

## 2 Primary and secondary mechanisms of resistance to EGFR-TKIs in NSCLC

One of the most frequent distinctive outcome of NSCLC patients is linked to the activation of *EGFR* mutations. Somatic mutations at exons 19-21, codifying for the tyrosine kinase domain, actually represent the main molecular condition to predict a good EGFR-TKIs response in upfront therapy (17, 18). First-generation TKIs, erlotinib and gefitinib, can compete in a reversible manner with adenosine triphosphate (ATP) at EGFR binding site, whereas the second-generation (e.g. afatinib, neratinib and dacomitinib) and third generation (e.g. osimertinib) TKIs can irreversibly block the ATP pocket of EGFR receptor, thereby inhibiting its phosphorylation and downstream signal transduction activity by covalently binding the ATP binding pocket mutations. As consequence, EGFR-TKI administration frequently allows a higher overall response rate (ORR) and progression-free survival (PFS) in *EGFR*-mutated metastatic patients compared with upfront chemotherapy (19, 20). In addition, osimertinib also received in recent years the approval for the administration in patients who acquired p.T790M mutation of *EGFR* as first/second-TKI generation resistance mechanism (21, 22).

In all cases, however, all therapies administered to inhibit oncogenic kinases activity are unable to completely eradicate tumors, so the *EGFR*-mutated patients invariably develop acquired resistance after 9-12 months of treatment initiation or they do not respond to TKIs treatment at all (23, 24). Several biological mechanisms of resistance have been reported to date, such as secondary *EGFR* mutations, bypass track signaling pathways and histologic transformation, not all strictly related to TKIs affinity (23, 25). All just reported mechanisms can be classified as primary or acquired resistance events, even if some of these, such as the co-expression of other ErbB receptors or the constitutive activation of other downstream pathways, remained ambiguous and are unlikely to be located in one of the two types of resistance.

Intrinsic or primary resistance refers to patients who either do not achieve stable disease or who progress within 6 months after an initial clinical response, according to the RECIST (Response Evaluation Criteria in Solid Tumor) criteria, with a consequent worsening of clinical conditions as well as response rate and disease control rates (LDCR), approximately in 20-30% EGFR-TKIs treated patients (26). Host-related mechanisms, such as defective immune system activity, rapid metabolism, or poor absorption, are predominantly responsible for intrinsic/primary resistances. Moreover, non-sensitive *EGFR* mutations, which contribute to an inconsistent drug activity, such as the naïve threonine-to-methionine substitution at the “gatekeeper” amino acid position 790 (p.T790M) in exon 20 and some mutations in exon 19 (p.L747S/p.D761Y), p.T854A or p.L868R in exon 21 of *EGFR*, can be included in this category (27, 28).

Apart from these, other molecular mechanisms could be the activation of different pathways by mutations in *HGF* (hepatocyte growth factor) gene (29), *IGF1R* (insulin growth factor 1 receptor) gene (30)*, MET* (MET proto-oncogene, receptor tyrosine kinase) gene (31), and/or *PI3K/AKT* (phosphatidylinositol-3-kinase and protein kinase B) pathway genes (32, 33). All above listed mechanisms of primary resistance generally arise after the administration of first- and second-generation TKIs in patients with NSCLC. There is also an emerging literature on primary resistance to the third-generation TKI osimertinib used in up-front therapy in *EGFR* mutated NSCLC, although data are actually in progress. The most compelling studies came from intrinsic resistance to osimertinib as second-line option: *KRAS* (Kirsten rat sarcoma virus) p.G12D mutation (co-occurring with the loss of *PTEN*, Phosphatase and tensin homolog, gene), *BRAF* (B-Raf Proto-Oncogene, Serine/Threonine Kinase mutation) mutations, *ALK* (Anaplastic lymphoma kinase) gene translocation, *HER2* (Human epidermal growth factor receptor-2) and *MET* (tyrosine-protein kinase Met) amplifications were reported (15, 34).

Acquired or secondary resistance to TKIs typically occurs in lung cancer patients after an initial response or stable disease to EGFR-TKIs (≥ 6 months), according to the RECIST criteria. In 50-60% of NSCLC patients who developed resistance to first/second-generation TKIs, the occurrence of p.T790M in exon 20 of *EGFR* is a fixed point for lung cancer management. In NSCLC patients with a pre-existent *EGFR* activating mutation, this last condition confers resistance to TKIs by sterically blocking the binding of drugs to the receptor pocket, thus giving an advantage to cancer cells by activating signaling pathways associated with tumor progression and metastasis (35). In this specific context, the administration of osimertinib as second line of treatment in patients harboring p.T790M mutation can re-block the tumor expansion, until additional resistance mechanisms occur as a result of the loss of the p.T790M mutation and the acquisition of novel resistance to second-line osimertinib, such as p.C797S mutation at exon 20 of *EGFR* (15, 34).

Other secondary mutations, in addition to the already mentioned p.T790M which are involved in EGFR-TKI acquired resistance, are represented by p.D761Y or p.L747S (exon19 of *EGFR*), and p.T854A (exon 21 of *EGFR*) (36). Uncommon and combined *EGFR* mutations, intratumoral heterogeneity beyond *EGFR* alterations, drug inefficacy due to adaptive mechanisms exploited by cancer to convey resistance, such as histological transformation of lung adenocarcinoma into small cell lung cancer (SCLC) (37), squamous cell carcinoma (SCC), as well as the activation of alternative pro-oncogenic signaling pathways are also reported (38). The epithelial-to-mesenchymal transformation (EMT) can be also included among these resistance mechanisms, as well as the consequent loss of cell adhesion and polarity and promote the formation of tumor stem cells and decreasing the EGFR signaling addiction (39).

**Figure 1:**
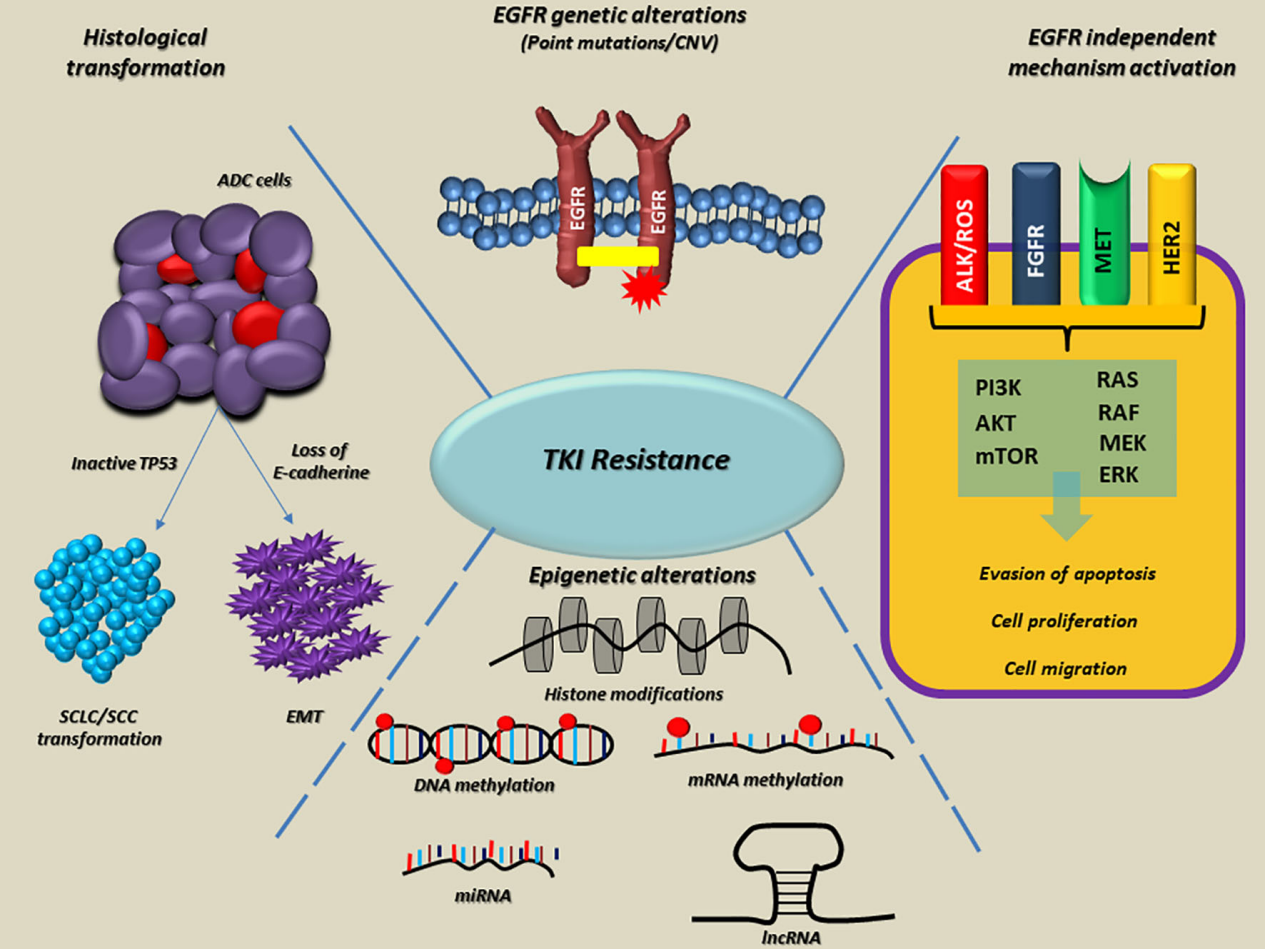
Overview of the main molecular mechanisms linked to EGFR-TKI resistance in NSCLC. On the top, *EGFR* alterations, as point mutations and copy number variations (CNVs) are involved both in intrinsic and acquired resistance to TKI. On the right side, the EGFR-independent mechanisms bypass RTK signaling through *ALK, ROS, FGFR, MET, HER2* alterations onset, thus promoting the activation of alternative and downstream pathways (e.g., PI3K/AKT/mTOR and RAS/RAF/MEK/ERK). At the bottom, epigenetic alterations such as histone modifications, DNA and RNA hypo/hypermethylation, aberrant expression of miRNAs and lncRNA frequently occur in several genes, which are able to promote tumor progression, metastasis and resistance to TKIs. Finally, on the left side, histological modifications such as SCLC or SCC transformation and EMT lead to the loss of sensitivity to EGFR TKIs in lung tumors. The dotted blue lines indicate interconnections among mechanisms linked to the EGFR-TKI resistance. EGFR, epidermal growth factor receptor; TKI, Tyrosine kinase inhibitors; Ex, exon; ins, insertion; amp, amplification; RTK, receptor tyrosine kinase; ALK, Anaplastic lymphoma kinase; ROS, ROS Proto-Oncogene, Receptor Tyrosine Kinase; FGFR, fibroblast growth factor receptor; MET, hepatocyte growth factor receptor; HER2, human epidermal growth factor receptor 2; PI3K, Phosphoinositide 3-kinase; AKT, Protein kinase B; mTOR, mammalian target of rapamycin; RAS, rat sarcoma virus; RAF, proto-oncogene c-RAF; MEK, Mitogen-activated protein kinase kinase; ERK, Extracellular signal-regulated kinase; miRNA, microRNA; lncRNA, long non-coding RNA; SCLC, small cell lung cancer; SCC, squamous cell carcinoma; EMT, epithelial−mesenchymal transition; ADC, adenocarcinoma; TP53, Tumor protein P53.

Variations in methylation levels and deregulation of miRNA and lncRNA machinery are widely associated with neoplastic transformation, carcinogenesis, and cancer progression. Anyway, the fluctuations of cancer methylome, both at DNA and RNA levels, remain the less investigated epigenetic changes in the context of target therapy resistance and TKI resistance in NSCLC (**Figure 1**).

## 3 The dynamic evolution of DNA methylation in TKI resistance of NSCLC: the state of art

**Figure 2:**
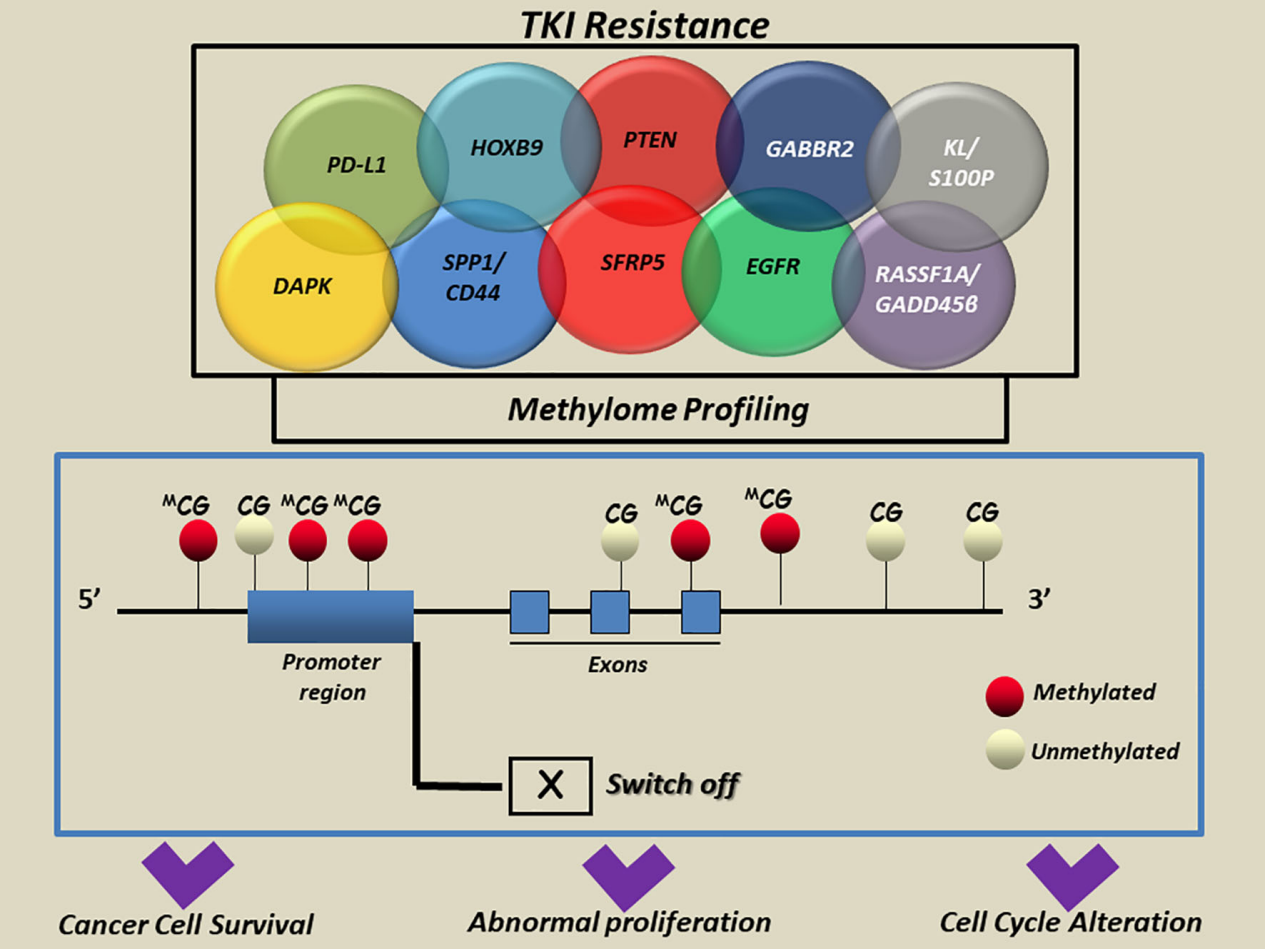
A simplified scheme of aberrant methylated genes associated with TKI resistance in NSCLC. Aberrant methylation of cytosine residues at the CpG islands in the promoter regions and/or interspersed CpGs mainly located in multiple genes was reported to modulate the transcription and induce aberrant regulation of genes implicated in cell-cycle alteration, abnormal proliferation and apoptosis escape in cancer cells. CG, cytosine guanine; DAPK, death-associated protein kinase; EGFR, Epidermal Growth Factor Receptor; GABBR2, Gamma-Aminobutyric Acid Type B Receptor Subunit 2; HOXB9, Homeobox B9; KL, Klotho; S100P, S100 Calcium Binding Protein; PD-L1, Programmed death-ligand 1; PTEN, Phosphatase And Tensin Homolog; RASSF1A, Ras association domain family 1 isoform A; GADD45β, DNA damage-inducible 45 beta; SFRP5, Secreted Frizzled Related Protein 5; SPP1, Secreted Phosphoprotein 1; CD44, cluster of differentiation 44; APC, adenomatous polyposis coli; BRMS1, Human breast carcinoma metastasis−suppressor 1; FOXA1, Forkhead Box A1; RARb, Retinoic Acid Receptor Beta; RASSF1A, Ras association domain family 1 isoform A; RASSF10, Ras Association Domain Family Member 10; SHISA3, Shisa Family Member 3; SLFN11, Schlafen Family Member 11; WIF-1, WNT Inhibitory Factor 1.

Starting from the molecular profiling of epigenetic marks across the genome, a new focus on the methylome evolution of lung cancer may help to more clearly understand how cell biology contributes to TKI drugs resistance in NSCLC patients (38, 40). The most interesting findings in this field are summarized in **Table 1** and detailed below. A representative scheme that depicts methylated genes and their associations with TKI resistance in NSCLC is also illustrated in **Figure 2**.

**Table 1:** Methylated genes and their functional and biological effects on acquired EGFR-TKI resistance in different lung cancer models.

Gene Symbol	Methylated region	Functional and biological effects	Detection methods	Cancer model	References
***EGFR***	49 CpGs of which six located in the promoter region (cg16751451, cg07311521, cg03860890, cg22396409, cg05064645, cg14094960). 43 CpGs along the gene body and in the 3’ UTR region.	✓ An inverse correlation between methylated CpGs of *EGFR* and mRNA/protein expression was observed. ✓ Promoter hypermethylation was found to be associated with immune cell infiltration and increased IFN-γ signature, while the opposite was found for methylation of the gene body region. ✓ Hypermethylation of cg02316066 and cg03046247 was strongly associated with lung adenocarcinoma prognosis.	Human methylation 450K array (TCGA dataset); qMSP	535 LUAD patients from TCGA and 20 paired LUAD/non-cancerous lung tissue samples.	(41)
***EGFR***	Promoter	✓ CpG island hypermethylation at the *EGFR* promoter enhances the sensitivity to gefitinib in NSCLC cells.	MSP	NSCLC cell lines: H1650, H1299 and PC-9.	(42)
***PTEN***	Promoter	✓ Aberrant methylation at promoter region may partially explain the lack of PTEN expression.	MSP	NSCLC cell lines and tissues from 125 patients with early-stage NSCLC.	(43)
***PTEN***	Promoter	✓ Hypermethylation of CpGs located at the *PTEN* gene promoter region inversely correlates with protein expression during acquired resistance to gefitinib or erlotinib. ✓ It enhances the Akt signaling pathway.	MSP	ADC, SqCC and SCLC cell lines (PC9, GEF1-1, GEF2-1).	(44)
***HOXB9***	cg13643585(enhancer region)	✓ Hypermethylation of cg13643585 in *HOXB9* correlates with intrinsic resistance to EGFR-TKI.	InfiniumHuman Methylation 450K array; Pyrosequencing	79 ADC from patients with *EGFR* mutations.	(45)
***PD-L1***	Promoter	✓ Promoter hypermethylation inversely correlates with expression levels. ✓ High methylation levels at the *PD-L1* promoter region are linked to the resistance to anti-PD-1 therapy in both chemotherapy or EGFR-TKI treated lung cancer patients.	Bisulfite sequencing	384 NSCLC patients divided in three sub-groups (EGFR wild type, n=214; EGFR p.L858R mutated, n=108; EGFR p.T790M mutated, n=62).	(46)
***GABBR2***	Exons 2 and 3	✓ High levels of *GABBR2* methylation at CpG islands negatively regulate GABBR expression and ERK1/2 pathway in NSCLC tumors and cell lines having *EGFR* 19 deletions.	MSCC sequencing Sequenom EpiTYPER	NSCLC cell lines (A549, HCC4006, HCC827) and lung ADC/non-neoplastic paired tissues from locally advanced stage IIIa patients.	(47)
***FRP5***	Unspecified CpGs	✓ Increased levels of methylation of *SFRP5* correlate to PFS reduction in NSCLC patients under EGFR-TKI treatment.	MSP	Tumor samples from 155 patients with stages IIIB to IV NSCLC, who received EGFR-TKI therapy.	(48)
***DAPK***	Promoter	✓ Hypermethylation of *DAPK* promoter induces gene silencing and is related to the acquired drug resistance in NSCLCs under erlotinib treatment.	MethDet-56 array qMSP	HNSCC and NSCLC (H226, SCC-1) cell lines treated with erlotinib/cetuximab.	(49)
***KL*** and ***S100P***	Interspersed CpG sites	✓ Promoter hypermethylation inversely correlates with expression levels. ✓ A possible role in acquired resistance to EGFR−TKI (gefitinib) is suggested.	Infinium Human Methylation 27 Bead Array	Human NSCLC cell line PC9 (EGFR exon 19 p.E746-A750del) and their gefitinib-resistant derivatives (PC9 GR, gr1, and gr3).	(50)
***SPP1*** and ***CD44***	cg00088885(*SPP1)* cg20971158 (*CD44)*	✓ CD44 and SSP1 methylation are prognostic factors in LUAD patients. ✓ SPP1 methylation modulates its expression and is related to 1st and 2nd EGFR-TKI resistance of NSCLC. ✓ CD44-SSP1 axis is implicated in the trasformation of ADC in SCLC under EGFR-TKI selettive pressure.	DNMIVD	GEO database (NSCLC): GSE122005: 3 resistant samples and 3 sensitive samples to gefitinib GSE31625: 28 samples and 18 sensitive samples to erlotinib; TKI-resistant NSCLC cell lines.	(51)
***RASSF1A*** and ***GADD45β***	Promoter	✓ Aberrant methylation at the promoter region of *RASSF1A* and *GADD45β* inversely correlates with protein expression and is linked to the acquisition of TKI resistance in lung cancer cells. ✓ Cell treatment with 5-Aza-CdR could partially restore the sensitivity of cells to EGFR-TKI.	NimbleGen Human DNA Methylation 3x720K Promoter Plus CpG Island Array; qMSP	Gefitinib-sensitive/resistant lung ADC cell lines (PC9, PC9/GR).	(52)
Nine gene set: ***APC, BRMS1, WIF-1, FOXA1, RARb, RASSF1A, RASSF10, SHISA3, SLFN11***	Promoter	✓ DNA methylation inversely correlates with mRNA expression in lung ADC tissues for all genes. ✓ Positive patients for almost one methylated marker showed a faster progression compared to negative patients for DNA methylation in all tested genes markers.	Data TCGA Research Network WBA; MSP	PB samples from 42 NSCLC patients.	(53)

NSCLC, Non Small Cell Lung Cancer; HNSCC, Head and neck squamous cell carcinomas; LUAD, lung adenocarcinoma; SCLC, small cell lung cancer; ADC, adenocarcinoma; SCC, squamous cell carcinoma; TKI, tyrosine kinase inhibitor; CpG, cytosine-phosphate-guanine; TR, Untranslated region; TCGA, The Cancer Genome Atlas; DMBs, differentially methylated blocks; MSP, methylation-specific PCR; qMSP, quantitative methylation-specific PCR; MSCC, Methyl-sensitive cut counting; DNMIVD, DNA methylation interactive visualization database; WBA, whole bisulfite amplification; PB, Peripheral blood. PFS, progression-free survival; GEO, Gene Expression Omnibus; DAPK, death-associated protein kinase; EGFR, Epidermal Growth Factor Receptor; MET, tyrosine-protein kinase Met; HER2, Human epidermal growth factor receptor-2; GABBR2, Gamma-Aminobutyric Acid Type B Receptor Subunit 2; CGI, CpG island; ERK1/2, extracellular-signal-regulated kinase; KL, Klotho; S100P, S100 Calcium Binding Protein P; PD-L1, Programmed death-ligand 1; PD-1, Programmed cell death protein 1; PTEN, Phosphatase And Tensin Homolog; 5-Aza-CdR, 5-aza-2′-deoxycytidine; HDAC, Histone deacetylases; RASSF1A, Ras association domain family 1 isoform A; GADD45β, DNA damage-inducible 45 beta; qPCR, Real-Time Quantitative PCR; SFRP5, Secreted Frizzled Related Protein 5; SPP1, Secreted Phosphoprotein 1; CD44, cluster of differentiation; BRMS1, Human breast carcinoma metastasis‑suppressor 1; FOXA1, Forkhead Box A1; RARb, Retinoic Acid Receptor Beta; RASSF10, Ras Association Domain Family Member 10; SHISA3, Shisa Family Member 3; SLFN11, Schlafen Family Member 11; WIF-1, WNT Inhibitory Factor 1; APC, adenomatous polyposis coli; IFN, interferon; HOXB9, Homeobox B9.

### 3.1 Methylated genes with STRONG evidence of association to TKI resistance in NSCLC

#### 3.1.1 EGFR

A new research front is opening up to explore a potential correlation between variation in methylation levels of specific CpG sites located at promoter and/or gene body regions of *EGFR* gene and response to EGFR-TKIs in lung cancer patients (14).

The first evidence of these links comes from *Li and colleagues’* studies. By performing *in vitro* assays on mutated (H1650 and PC-9) and wild-type (H1299) *EGFR* lung cancer cell lines, they showed that *EGFR* promoter hypermethylation enhanced the antitumor effect of TKI gefitinib and modulated the expression of EGFR both at transcript and protein level. Moreover, the resistance to gefitinib in unmethylated PC-9 tumor cells having *EGFR* exon19 deletion was bypassed by using a combined treatment of 5-aza-2’-deoxy cytidine (5-aza-CdR) and gefitinib, thus boosting the growth inhibitory effects and leading to the activation of caspases (42).

The most significant support to this first evidence came from the integrative multi-omics analysis by *Xu Z and colleagues*, who investigated the *EGFR* genes in terms of CpGs methylation (49 CpG sites), somatic mutations, copy number variations (CNVs), transcriptional and protein expression level fluctuations in 535 lung adenocarcinoma (LUAD), available from The Cancer Genome Atlas (TCGA). A large number of *EGFR* CpGs located at the promotor region was identified by Illumina HumanMethylation450K and RNA-seq data analysis, whose methylation levels showed an inverse correlation with the transcription level variations, protein expression and CNVs of *EGFR* gene. By contrast, the aberrant methylation of CpGs located at the gene body regions was found to positively correlate with the EGFR protein expression. In LUAD patients having *EGFR* mutations, the Authors found that most CpG sites were hypomethylated and about 30% of these were predictive of a good prognosis for patients. In addition, promoter *EGFR* hypermethylation was found to be associated with the immune cell infiltration and increased IFN-γ signature, while an opposite correlation was found for methylation of the CpGs at gene body region. Finally, the hypermethylation of cg27637738, cg16751451, cg02316066, cg22396409, cg03046247, cg02166842, cg21901928, cg07311521 and cg06052090 CpG sites was associated to a poor prognosis in lung adenocarcinoma patients (p<0.05). In particular, cg02316066 and cg03046247 were the most strongly associated ones and showed a high degree of co-methylation with cg02316066 and cg03046247 (p<0.001) (41).

#### 3.1.2 PTEN

*PTEN* (phosphatase and tensin homolog) is a lipid phosphatase that is involved in the negative regulation of phosphatidylinositol 3-kinase (PI3K)-AKT signaling and radio-chemotherapy in tumors. Genetic aberrations of *PTEN* are not frequent in NSCLC; by contrast, in about 35% of early stage NSCLC samples the lack of PTEN protein expression observed was reported to correlate with hypermethylation at the promoter gene region. Moreover, *PTEN* aberrant methylation was observed in NSCLC cell lines and was correlated to transcript and protein level fluctuations under *in vitro* treatment with the 5-aza-2’-deoxycytidine (43).

Consistent with data by *Soria and colleagues*, Maeda and his team explored a possible correlation between hypermethylation of CpGs located at the *PTEN* gene promoter region and resistance to gefitinib or erlotinib in the two lung cancer cell sublines GEF1-1 and GEF2-1 (obtained from cell line harboring the *EGFR* mutation p.E746_A750del). It was observed that the region located 329 to 124 nucleotide upstream from the translation initiation site of the *PTEN* promoter region was hypermethylated only in resistant cell lines. This condition inversely correlated with PTEN protein expression. *PTEN* suppression enhances the AKT phosphorylation, thus switching on the expression of cyclin D1 and ICAM-1 (intracellular adhesion molecule-1) and accelerating the migration of the cancerous cells (54). These evidences support the suggestion of an alternative approach for TKIs in combination with demethylating 5-aza/HDAC (Histone deacetylases) inhibitors or Trichostatin A (TSA) to hinder lung tumor growth, whose efficacy was observed on gefitinib-resistant PC9/f9 and PC9/f14 cells by Noro T et al. (44).

#### 3.1.3 HOXB9

*HOXB9* (Homeobox B9) gene codified for a sequence-specific transcription factor that is implicated in several processes from cell development to cell proliferation by enhancing the EMT, the expression of angiogenic factors (VEGF, IL-8, and/or TGFβ), and *EGFR* and *ErbB2* pathways activation, through the AKT/NF-κB/Snail pathway (55). The effects of epigenetic *HOXB9* regulation on intrinsic and acquired TKI resistance in NSCLC patients are actually debated. A possible correlation between methylation profiling of 216 CpG sites (islands and S-shores) by Illumina Infinium Human Methylation 450K array and gene expression profile was investigated in stage III and IV *EGFR*-mutated NSCLC patients. A critical role of cis-regulation of expression by methylation in lung adenocarcinoma and intrinsic resistance to EGFR-TKIs were found both in the discovery (79 tumors sampled from patients with advanced lung adenocarcinoma before receiving EGFR-TKI) and in the validation cohort (163 patients with *EGFR*-activating mutations) of NSCLC patients. Specifically, *HOXB9* aberrant methylation at cg1364358 site, located in the enhancer region of gene, was found to be strictly related to disease progression of patients after TKI treatments and to disease monitoring, since it was able to predict a disease control rate with 88% sensitivity in patients having *EGFR* activating mutations (45).

In addition to these evidences, novel genome-wide studies using liquid biopsy samples of 122 NSCLC patients under erlotinib (67.2%), gefitinib (11.5%) or afatinib (2.5%) treatment demonstrated a correlation between hypermethylation of regulatory regions of *HOX* genes and TKI resistance in those patients also having *MET* or *HER2* amplifications. Such consistent findings indicated that the hypermethylation status of *HOX* genes could be exploited not only to monitor EGFR-TKI resistance in NSCLC patients, but also to predict and treat *MET* or *HER2* amplification mediated resistance (56).

### 3.2 Methylated genes with LOW evidence of association to TKI resistance in NSCLC

#### 3.2.1 PD-L1

Z*hang Y et al.* gave an interesting indication about the role of *PD-L1* (programmed cell death ligand 1) promoter aberrant methylation in mediating the mechanisms of resistance to the anti-PD1 treatment in *EGFR* mutated NSCLC patients. A total of 384 surgical NSCLCs, previously profiled for the *EGFR* mutation status (three groups as follows: wild-type group, n=214; p.L858R group, n=108 and p.T790M group, n=62) were tested by PCR bisulfite sequencing to measure the ratio of CpGs methylation level at the *PD-L1* gene promoter region. After cancer recurrence, the PD-L1 was found to be up-regulated in patients treated with chemotherapy or EGFR-TKI therapy, but decreased in the patients with anti-PD1 therapy. Promoter methylation analysis showed that the secondary NSCLC after cancer recurrence with anti-PD1 therapy had higher levels of *PD-L1* methylation compared to those naive cancers and/or normal tissues (46).

The *in vivo* experimental validation performed by the same Authors in mice model showed that the increase of *PD-L1* promoter methylation levels reflected the reduction in PD-L1 expression after nivolumab therapy, irrespective of *EGFR* mutation status. This may be due to a pre-existent heterogeneity in *PD-L1* methylation patterns in tumor cells or to a tumor cell evolution and switch-off the PD-L1 expression through epigenetic modulation induced by the selective pressure of the drug. Anyway, both hypotheses remain unconfirmed and need to be supported by further investigations (46).

#### 3.2.2 GABBR2

*GABBR2* (Gamma-Aminobutyric Acid Type B Receptor Subunit 2) gene encodes a multi-pass membrane protein that belongs to the G-protein coupled receptor 3 family and GABA-B receptor subfamily. The GABA-B receptors inhibit neuronal activity through G protein-coupled second-messenger system, which regulates the release of neurotransmitters, and the activity of ion channels and adenylyl cyclase (57). The role of GABBR2 in cancer progression was firstly supposed in thyroid carcinomas, but an elevated expression of this gene was also reported as a specific feature of lung cancerous lesions and linked to a better prognosis of patients (58, 59).

The first suggestion of a correlation between DNA methylation of *GABBR2* and resistance to TKI erlotinib in NSCLC patients comes from the study by *Niu X et al.* who investigated the variations of methylation patterns by whole-genome DNA high-throughput assays in a small cohort of NSCLC patients under erlotinib treatment (47). Specifically, the epigenetic profile of *GABBR2* gene at promoter region prior to and following erlotinib treatment were compared in two IIIa stage NSCLC patients having *EGFR* activating mutations (exon19 p.E746-A750del and p.A750-E758del). As result, the same differentially methylated region (DMR), located between exon 2 and exon 3 of *GABBR2* gene, was found in both patients, with an average of methylation changes of 42.35% and 23.50% in the two patients, respectively. Lung cancer tissues of patients tested by IHC before and after induction to erlotinib treatment in the two patients showed a consistent decrease of GABBR2 expression after erlotinib treatment. The following *in vitro* experiments also demonstrated a direct role of erlotinib in *GABBR2* methylation and the consequent downregulation of its expression in *EGFR*-mutated lung tumor cells. Conversely, the upregulation of GABBR2 may restore TKI-induced cell apoptosis through ERK1/2 and its crosstalk pathway signaling.

Taking all together, the above findings provide a new theoretical basis for expanding this epigenetic investigations to a more large cohort of *EGFR*-mutated NSCLCs and suggest a possible role of *GABBR2* in improving clinical outcomes of TKI treated patients with locally advanced NSCLC (47).

#### 3.2.3 SFRP5

*SFRP5* (Secreted Frizzled Related Protein 5) gene codifies for one of the soluble Wnt signaling modulators that are involved in the regulation of cell proliferation and cancer progression (60, 61). *Zhu J and colleagues* reported that hypermethylation of *SFRP5* gene was able to predict a worse outcome in EGFR-TKI advanced adenocarcinoma patients. In their study, the Authors quantified the DNA methylation levels of a selected group of Wnt antagonist genes, after the administration of EGFR-TKI in a cohort of 155 NSCLCs of IIIB to IV patients. The correlation between the methylation status of *SFRP5* gene prior to treatment and progression-free survival (PFS) showed that the Wnt antagonist genes tend to be simultaneously methylated, as well as methylation of *SFRP5* reversely correlated with *EGFR* mutation status of patients (p= 0.011). Moreover, the subgroup of TKI-treated patients with higher *SFRP5* methylation levels showed a worse OS and PFS compared to the group with low or absent *SFRP5* methylation, independently from the *EGFR* genotype.

Finally, it was observed that patients without methylation in *SFRP1* have a longer PFS compared with patients with its methylation (9.7 months *vs* 2.0 months, p = 0.05), thus suggesting the intriguing hypothesis that activation of Wnt signaling by antagonist methylation could confer staminal properties linked to the EGFR TKIs resistance in lung cancer patients (48).

#### 3.2.4 DAPK

DAPK (death-associated protein kinase) protein belongs to the calcium/calmodulin (CaM)-regulated serine/threonine protein kinase family with pro-apoptotic function through the interferon-γ, TGF, TNFα and Fas ligand mediators (62–64). Variations in DAPK expression are observed in NSCLC and in many other cancer types, at times due to DNA hypermethylation at the promoter region (65, 66).

The link between *DAPK* (death-associated protein kinase) promoter methylation and acquired resistance to anti-EGFR TKIs was investigated for the first time by *Ogawa T and collaborators* in a collection of cancer cell lines. To experimentally validate their hypothesis, a 56 genes panel methylation (MethDet-56) array was used to assess differences in methylation profile of a collection of head and neck squamous cell carcinoma (HNSCC) and NSCLC cell lines before and after acquired resistance to erlotinib and cetuximab treatments. An hypermethylation of *DAPK* gene at the promoter region linked to a decrease in its expression was exclusively observed in resistant lung cancer lines of both types of drugs and not in the parental cells. Taking into account that *DAPK* appeared to be silenced in HNSCC cells through DNA methylation long before the treatment with EGFR inhibitors, the Authors suggested *DAPK* as an epigenetic mediator in acquired resistance only in NSCLC but not in HNSCC cells (49). Even if of great interest, further studies are demanded to corroborate these results on tumor samples from NSCLC patients under TKI treatment.

#### 3.2.5 *KL* and *S100P*

To clarify the role of epigenetic regulatory mechanisms in the resistance to gefitinib, *Terai H et al.* compared variations of global DNA methylation profile in gefitinib-sensitive and resistant lung cancer cell lines. The comprehensive DNA methylation and mRNA expression analyses performed allowed the identification of 640 genes associated with secondary resistance to EGFR-TKI. Among these, experiments of silencing by siRNA and 5-aza-dC treatment highlighted the potential role of methylation in *KL* (Klotho) and *S100P* (S100 Calcium Binding Protein P) genes in the acquisition of resistance to gefitinib (50). Anyhow, no additional studies on patients ‘cohorts were conducted to date.

#### 3.2.6 *SPP1* and *CD44*

*SPP1* (Secreted Phosphoprotein 1) encodes the osteopontin (67), which has been found to abnormally express in a variety of cancers, and induces drug resistance, progression, recurrence, and metastasis in breast, ovarian, and colon cancer (68–70). Together with *CD44* (cluster of differentiation), it also contributes to early pathogenesis and metastatic potential in lung cancer (71).

Both *SPP1* and *CD44* genes were suggested by *Wang et al.* as molecular drivers that might contribute to EGFR-TKI resistance in NSCLC (51).

This interesting scientific evidence resulted from the evaluation of the transcriptional activity of the two genes in resistant NSCLC samples EGFR-TKI treated of GEO (Gene Expression Omnibus) database, which was found to be increased *versus* the sensitive ones. Data analysis on resistant NSCLCs to the 1st or 2nd TKI generation revealed that high methylation of CpGs at *SPP1* (cg00088885) and *CD44* (cg20971158) promoters could be considered a potential, independent indicator of the worst prognosis in lung adenocarcinoma patients. The upregulation of SPP1 due to hypermethylation induces resistance to the 1st and 2nd generation EGFR-TKI and influences tumor immune infiltration in tumor tissues and cell lines. Moreover, co-expression studies revealed that SSP1-CD44 axis deregulation identifies the same group of miRNA involved in transforming NSCLC into SCLC mediated by a multidrug-resistant cancer stem cells acquisition, thus modulating the cancer phenotypes transition in acquiring resistance to TKI process (51).

#### 3.2.7 *RASSF1A* and *GADD45β*

*RASSF1A* (Ras association domain family protein1 isoform A) encodes for a tumor suppressor protein that exerts several anti-tumoral effects by modulating tumor growth and dissemination through several biological functions as well as cell cycle arrest, migration inhibition, and/or apoptosis induction (72). The *GADD45β* (Growth Arrest And DNA Damage-Inducible Protein GADD45 Beta) belongs to a list of genes involved in stressful growth arrest conditions and treatment with DNA damaging molecules (73). *Hou and colleagues* assessed the methylation status at the promoter region of *RASSF1A* and *GADD45β* genes in acquired gefitinib-resistant lung adenocarcinoma PC9 (harboring *EGFR* exon19 deletion) and PC9/GR (harboring *EGFR* exon19 deletions and acquired *EGFR* exon20 p.T790M mutation) cell lines. Using Nimble Gen Human DNA Methylation 3x720K Promoter Plus CpG Island Array, they found that the promoter regions of both genes were hypermethylated only in PC9/GR cell line and that the epigenetic silencing induced by methylation was able to induce a downregulation of RASSF1A and GADD45β expression. To confirm the link between the observed methylation in both genes and resistance to gefitinib in PC9/GR cells, the Authors showed that this process could be partially reversed by using the demethylating agent 5-Aza-CdR (52). No confirmative studies on patients ‘cohorts were conducted to date.

### 3.3 Multi-gene and Genome-wide global methylation profile and EGFR-TKI resistance

Increasing evidences about the role of DNA methylation in the molecular pathology of lung cancer highlights the need for robust technologies able to establish if whole methylome, and not only methylation changes in single or few genes, could be associated with EGFR-TKI resistance (9, 74). To date, multiple high-throughput techniques are available for assessing genome methylation and determining DMRs, both at tissue and at liquid biopsy levels, for real-time monitoring of disease load in advanced lung cancer patients (8, 75–77). Despite this, a few original studies underlined the association between whole methylome fluctuations and therapeutic resistance to first/second/third-TKI treatments in NSCLC patients.

One of the earliest evidence was provided by *Xia S and colleagues*’ work, which reported that a concomitant evaluation of molecular profile and whole methylation status in lung tumors was useful to early predict response to second-line osimertinib in NSCLC patients (78). Plasma samples from n=8 stage IV osimertinib-treated *EGFR* p.T790M-positive patients with lung adenocarcinoma were longitudinally collected and analyzed using capture-based targeted DNA and methylated DNA sequencing. A significant inverse correlation between allele fraction rate and methylation status was observed (p=0.0002), which was absent in those patients who did not have any somatic mutations (78).

The link between DNA methylation profile and TKI treatment in NSCLCs was also reported in a prospective study on 36 *EGFR* mutant NSCLC patients. Tumor tissues were obtained from 10 patients prior to the TKI treatment, of which 4 matched with post-TKI re-biopsies (3 p.T790M+, 1 p.T790M-). The remaining tissues from post-TKI patients were divided into 17 positive and 9 negative for p.T790M mutation groups. The epigenetic profiling by Illumina Infinium EPIC array allowed the identification of a correlation between *EGFR* p.T790M and the epi-methylated group of patients. All post-TKI p.T790M+ samples fell within epi-group 2, whereas most of p.T790M- samples were found within epi-group 1. The report suggested that the acquisition of resistance to EGFR-TKIs could already occur at baseline and could be related to a specific multigene DNA methylation pattern, which includes probes mapping in the *EGFR* gene (79).

*Nguyen HN and colleagues* published a fascinating study suggesting how TKI resistance mechanisms could be associated with important changes in epigenetic profiles of lung tumors. The Authors tested plasma cell-free DNA (cfDNA) samples of 122 Vietnamese advanced NSCLC patients at stage III or IV of the disease, who had a clinical story of acquired resistance, following gefitinib/erlotinib or afatinib treatment. Using ultra-deep massively parallel sequencing targeting 450 genomic regions and covering 9593 CpG sites in nine genes (*EGFR, KRAS, NRAS, BRAF, ALK, ROS1, MET, HER2* and *PIK3CA*), it was observed that the heterogeneity of methylation patterns occurred in those cases that have different mutation profiles of acquired resistance to TKI drugs (56). Of those, genetic alterations in *EGFR*, particularly *EGFR* amplification (n = 6), showed an associated genome-wide hypomethylation. Interestingly, the level of hypomethylation was associated with the duration of response to TKI treatment.

Novel important preliminary evidences came from *Shi and colleagues* investigations, who constructed a DNA methylation-based risk score (RS) to better predict survival in *EGFR* mutated NSCLC patients after TKIs treatment. Forty mutated (Ex19del or L858R) and 21 *EGFR* wild-type blood samples of NSCLC patients were profiled by targeted bisulfite sequencing using a panel with 80,672 CpG sites, covering more than 1 million bases of the human genome. A total of 56 differentially methylated blocks appeared to be significantly downregulated in *EGFR* mutated group under TKI treatment. A four-DMB based prognostic RS model involving 4 cancer-related genes was developed to predict poor PFS, independently from their clinical factors (p<0.001) (80).

A recent study conducted by *Ntzifa A et al.* suggested that variations in DNA methylation levels of a group of nine genes (*RASSF1A, RASSF10, APC, WIF-1, BRMS1, SLFN11, RARβ, SHISA3 and FOXA1*) may play a direct role in resistance to osimertinib as second line treatment. Eighty cell-free DNA samples and 74 circulating paired tumor cells (CTCs) were collected from a total of 42 NSCLC patients, before osimertinib treatment and at the time of disease progression. The Authors proved a direct and strong correlation between *RASSF1A* and *APC* methylation levels at promoter regions. In addition, methylation rates of *APC*, *WIF-1* and *SLFN11* were found to be higher at PD. Positive NSCLC patients for at least one methylated marker had a more rapid progression than the full negative ones.

Although it was not evident a correlation between methylation and PD comparing cfDNA and paired CTC groups in 42 NSCLC patients, a concordance trend was found for 6 methylated genes (*APC, BRMS1, RASSF1A, RASSF10, SLFN11, WIF-1*). More interestingly, positive patients for almost one methylated marker showed a faster progression than patients negative for DNA methylation for all tested markers (p=0.031) (53).

## 4 Role of miRNA in EGFR-TKI resistance-brief overview

MicroRNAs (miRNAs) are 18-25 nucleotides single-stranded non-coding RNAs which are able to turn on and off the expression of their targeted genes (81). The deregulation of miRNA machinery is linked to the acquisition of cancer hallmarks that lead to genome instability and impact on tumor growth, invasion and metastatization (82). Recent findings have shown that miRNAs could modulate as post-transcriptional regulators the response to EGFR TKIs in overcoming resistance in NSCLC patients, as summarized in **Table 2** (92, 93). The first evidence of this derived from *Hashida S and colleagues’* work, whom investigated the EGFR-TKI resistance mechanisms in a total of 10 afatinib-resistant cell lines from parental NSCLC cells with activating *EGFR* mutations (83). In particular, they found that these *EGFR*-mutant lung cancer cell lines mainly acquired *MET* amplification as a mechanism of resistance and become sensitive to afatinib plus crizotinib. The acquired *MET* amplification was co-occurrent with miR200c epigenetic silencing and the acquisition of EMT and stem cell-like features. Moreover, it was demonstrated that the acquisition of EMT and other associated features was also due to a downregulation of epithelial markers as well as E-cadherin that was observed in this group of afatinib-resistant cell lines. These findings were consistent with previous observations by *Shien et al.* (84), who reported a downregulation of miR200c by methylation in a group of NSCLC cell lines gefitinib resistant having acquired *MET* amplification and stem cell-like features (83).

**Table 2:** Functional and biological role of miRNAs related to EGFR-TKI resistance in lung cancer models.

miRNA	Functional and biological effects	Cancer models	Refs
**miR-200c**	✓ miR200c epigenetic silencing and downregulation co-occurred with *MET* amplification in *EGFR*-mutant cells after acquired afatinib resistance and makes them more sensite to afatinib plus crizotinib. ✓ miR200c epigenetic silencing are related to the acquisition of EMT and stem cell-like features in the afatinib-resistant lung cell lines.	✓ Afatinib-resistant lung cell lines obtained from parental NSCLC cells with activating *EGFR* mutations.	(83)
**miR-200c**	✓ miR200c hypermethylation was associated to acquired resistance to gefitinib in lung cancer cells having *MET* amplification by promoting EMT features. ✓ miR200c epigenetic silencing are related to the acquisition of stem cell-like features in the gefitinib-resistant lung cell lines.	✓ *EGFR*-mutant lung cell lines and sublines resistant to gefitinib.	(84)
**Let-7c **	✓ Let-7c acts as a regulator of EMT as well as affects CSC phenotype. ✓ Its expression was correlated with resistance to osimertinib in p.T790M NSCLC cells.through the EMT modulation.	✓ H1975 (endogenous p.T790M mutation) and HCC827-T790M (with acquired p.T790M mutation) lung cancer cell lines.	(85)
**miR-7**	✓ miR7 enhances gefitinib cytotoxicity by suppressing both *EGFR* and *IGF1R* signaling.	✓ Adenocarcinoma lung cell (A549).	(86)
**miR-130a**	✓ miR-130a overexpression enhanced apoptosis and suppressed NSCLC cells proliferation before and after gefitinib treatment via MET signaling. ✓ Overexpression of MET could rescue the functions of this miRNA regarding cell apoptosis and proliferation after treatment with gefitinib.	✓ Gefitinib-sensitive and resistant NSCLC cell lines.	(87)
**miR-200a**	✓ miR-200a is downregulated in NSCLC cells, where it directly targets the *3′-UTR* of both *EGFR* and *MET* mRNA. ✓ Its overexpression significantly downregulates both *EGFR* and *MET* signaling pathways and severely inhibits cell migration, invasion in gefitinib resistant lung cells.	✓ Gefitinib-sensitive and resistant NSCLC cell lines.	(88)
**miR-133b**	✓ The increase of miR-133b expression led to a decrease in lung cancer cell growth. ✓ Variations in its expression could help to discriminated responder from non-responder patients to TKI erlotinib. ✓ High levels of miR-133b in NSCLCs were associated with longer progression-free survival time of NSCLC patients.	✓ NSCLC cell lines (A549 and H1299) and 32 patients with advanced lung ADC who received erlotinb as second- or third-line therapy.	(89)
**miR-497**	✓ miR-497 overexpression can reverse drug resistance of NSCLC cells to EGFR-TKI by inhibiting the expression of IGF1R protein and blocking the activation of its downstream *AKT1* signaling pathway.	✓ Gefitinib resistant lung adenocarcinoma A549 cell line (A549/GR).	(90)
**miR-30a-5p**	✓ Gefitinib combined with miR-30A-5p mimics, could suppress the growth in acquired TKI resistance lung cancer cells via IGF1R and HGFR signaling.	✓ H1650-acquired gefitinib-resistant cell (H1650GR), H1975, and H460 cell lines.	(91)

miRNA, microRNA; MET, MET Proto-Oncogene, Receptor Tyrosine Kinase; EGFR, epidermal growth factor receptor; EMT, epithelial-to-mesenchymal transition; CSC, Cancer stem cells; IGF1R, Insulin-like growth factor1 receptor; AKT, Protein kinase B; PI3K, phosphoinositide 3-kinase; ADC, adenocarcinoma; NSCLC, Non Small Cell Lung Cancer.

Many other reports are providing evidences about the contribution of miRNAs in the complex and heterogeneous mechanisms of EGFR-TKI drug resistance in NSCLC. A role in EMT promotion linked to acquired osimertinib resistance in NSCLC was also attributed to Let-7c (85), whereas miR-7 was demonstrated to enhance TKI-induced cytotoxicity by gefitinib through the suppression of both *IGF1R* (Insulin like growth factor1 receptor) and *EGFR* signaling pathways (86).

A critical role of miR-130a in overcoming acquired resistance to EGFR-TKIs via *MET* signaling was highlighted in two separate studies. A first study demonstrated that the overexpression of miR-130a enhanced apoptosis and suppressed NSCLC cells proliferation after gefitinib treatment. Otherwise, a down-regulation of this miRNA triggered cell apoptosis with rapid proliferation in both gefitinib-sensitive and resistant NSCLC cell lines. It was also demonstrated that miR-130a binds to the *3’-UTR* of *MET* and significantly suppresses its expression, so the overexpressing of MET could rescue the functions of miR-130a regarding cell apoptosis and proliferation after cells are treated with gefitinib (87). These results are in line with those published in 2015, when it has been shown that the decrease of cell invasion and migration due to miR-200a overexpression leds to gefitinib resistance in NSCLC cells via *EGFR* and *MET* signaling (88).

*Bisagni A et al.* was able to prove how the increase expression levels of miR-133b significantly correlated with a better PFS and OS and allowed to better discriminate NSCLC responder patients to erlotinib from non-responder. Similarly, they found a direct relationship between the upregulation of miR-200c and improved gefitinib sensitivity in NSCLC (89).

Finally, increased miR-497 level was seen to have a significant impact in enhancing sensitivity to EGFR-TKI in NSCLC cells via *IGF1R* targeting and *AKT* activation (90), whereas *in vitro* experiments and *in vivo* models were useful to demonstrate that the combination of gefitinib plus a microRNA mimic, miR-30a-5p, could overcome acquired EGFR-TKI resistance in NSCLC via a direct regulation of *IGF1R* and *HGFR* (hepatocyte growth factor receptor) signaling (91).

## 5 Concluding Remarks

Worldwide, lung cancer represents one of the most common types of cancer and, by far, the leading cause of cancer deaths (94, 95). Among these cases, 80-85% have an NSCLC histology, which includes adenocarcinoma, squamous cell carcinoma, and large cell carcinoma subtypes (96, 97). Changes in DNA methylation may largely underpin lung cancer in several processes as well as the capability of growth, invasion and spreading of cancer cells (98, 99). A special attention is now drowning toward DNA methylation patterns of drug-treated tumor cells that could change and support the acquisition of resistance to treatments.

The double interaction between epigenetic alterations and therapy resistance of tumors is progressively emerging and is looking for answers as to why it happens. In solid tumors and in EGFR-TKI treated NSCLC patients there are little translational evidences about the occurrence and role of DNA methylation at different regions of single/multiple genes (promoter and other regulatory regions) or CpG islands (14, 100).

To bridge the gap in this specific field is actually demanded. Specifically, it is necessary to understand how methylome could be modified and which methylated regions or specific CpG sites are affected in order to translate relevant epigenome associations into clinically personalized treatment in lung cancer patients (101). Next to this, the definition of methodological high-throughput approaches to study changes in methylation will improve the advances in this field, since measuring global or single CpG methylation will help to construct a more robust and integrated algorithm to predict and monitor disease evolution in a non-invasive manner for lung cancer patients (102–104). In recent years, there has been a growing interest in liquid biopsy to identify and monitor epigenomic drivers, also in the context of primary and acquired resistance in lung cancer (105). There are many reasons why circulating tumor DNA (ctDNA) methylation may rapidly emerge in this clinical settings. The first point leads to the fact that DNA methylation occurred as an early event in the etiology and progression of lung carcinogenesis, besides it could be strictly dependent both on tissue location and type of cancer (106). Second, it should be taken into consideration that DNA methylation profiling provides a deep characterization of ctDNA which contains important information about longitudinal changes in CpG islands across genomic regions (107). Third, the heterogeneity of CpG methylation patterns in different regions of multiple genes (promoter and other regulatory regions) is significantly associated with a poor outcome that might allow for accurate discrimination among lung cancer subtypes in liquid biopsies samples (108). Moreover, starting from the track of tumor evolution in serial ctDNA, it could be possible to identify minimal residual disease and manage early cancer progression, overcome temporally and spatially intratumor heterogeneity aiming at stratifying lung cancer patients according to recurrence risk and response to therapy (109). Although it remains to define several methodological strategies as well as optimize ctDNA extraction step to ensure a high-quality cfDNA or establish the gold standard for setting a better sensitivity and specificity of ctDNA methylation assay detection (110), a systematic analysis of liquid biopsy samples could provide important insights into the heterogeneity of TKI resistance mechanisms in NSCLC patients, thus providing essential information to better predict resistance and help the selection of subsequent treatments.

Ongoing research studies are also focused on single-cell DNA-methylation profiling that may contribute to the examination of cell-of-origins and cancer cell type heterogeneity by which becomes possible to clarify the correlation between DNA methylation and the expression fluctuations of cancer driver genes sets in different subtypes.

## Author contributions

Conceptualization, FPF and LM. Data curation, FPF and AS. Writing—original draft preparation, FPF. Writing—review and editing, LM. Visualization, AS. Supervision, LM. All authors contributed to the article and approved the submitted version.
